# Complete Radiologic Response in Metastatic Castration-Resistant Prostate Cancer Treated with Cabazitaxel

**Published:** 2015-05

**Authors:** Bahram Mofid, Samira Azghandi, Ahmad Rezazadeh Mafi

**Affiliations:** 1 Clinical Oncologist, Shohada Hospital, Shahid Beheshti University of Medical Sciences, Tehran, Iran;; 2 Clinical Oncologist, Imam Hossein Hospital, Shahid Beheshti University of Medical Sciences, Tehran, Iran

## Dear Editor,

About 20-40% of patients who undergo primary therapy for early-stage prostate cancer, experience biochemical relapse of which 30-70% develop metastatic disease within 10 years^1^ and the majority will become resistant to castration (CRPC). Since 2004, docetaxel has been the first-line cytotoxic treatment in treating metastatic CRPC. However, many mCRPC patients eventually become resistant to docetaxel, for whom available treatment options are limited.^2^ Cabazitaxel, a next-generation taxane, is the first agent to demonstrate improved survival post-docetaxel in mCRPC patients.^3-5^

**Figure 1:**
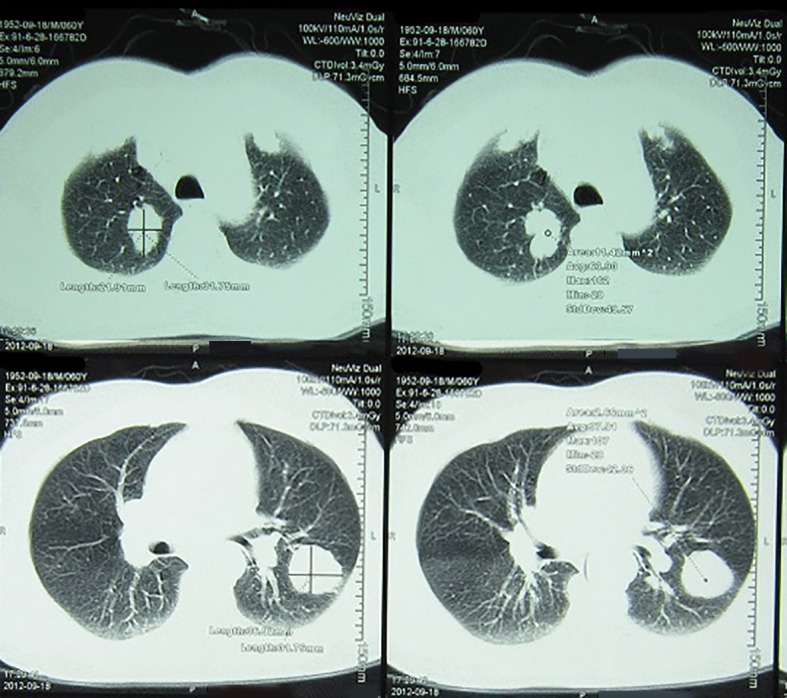
Shows the chest CT scan of the patient before starting chemotherapy with docetaxel.

In 2007, a 62-year-old patient presented to our clinic with a PSA=6.5 ng/ml. He refused a biopsy, and returned in August 2009 with PSA=19 ng/ml. Prostate biopsy was done in September 2009, followed by radical prostatectomy. The pathology report came back as prostate adenocarcinoma with Gleason 3+4, extensive PNI, and involvement of both lobes as well as the right seminal vesicle (pT3). Iliac lymph nodes were free of tumor. After the surgery, PSA reduced to 0.1 ng/ml and he was planned to receive radiation therapy to the prostate bed followed by monthly decapeptyl. In November 2011, while still on androgen deprivation therapy with PSA=0.05 ng/ml, he developed dyspnea and hemoptysis. A chest CT scan revealed several masses in both lungs and metastasis was proved by biopsy (figure 1).

Chemotherapy with docetaxel and prednisolone was started. After the 6^th^ cycle, CT scan showed a significant reduction in size and number of lesions, and chemotherapy continued for two more cycles.

**Figure 2:**
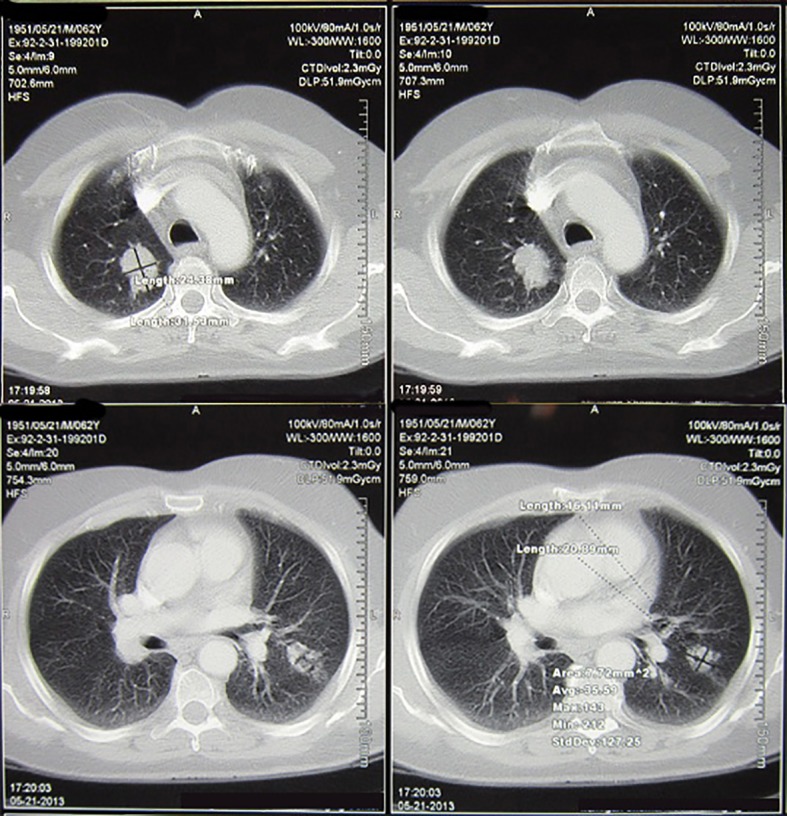
Shows the chest CT scan of the patient 3 weeks after the 8th cycle of chemotherapy with docetaxel.

Six weeks after the 8^th^ cycle of chemotherapy, he complained of dyspnea again, and at that time, the chest CT scan revealed an increase in the size and number of pulmonary lesions (figure 2).

**Figure 3:**
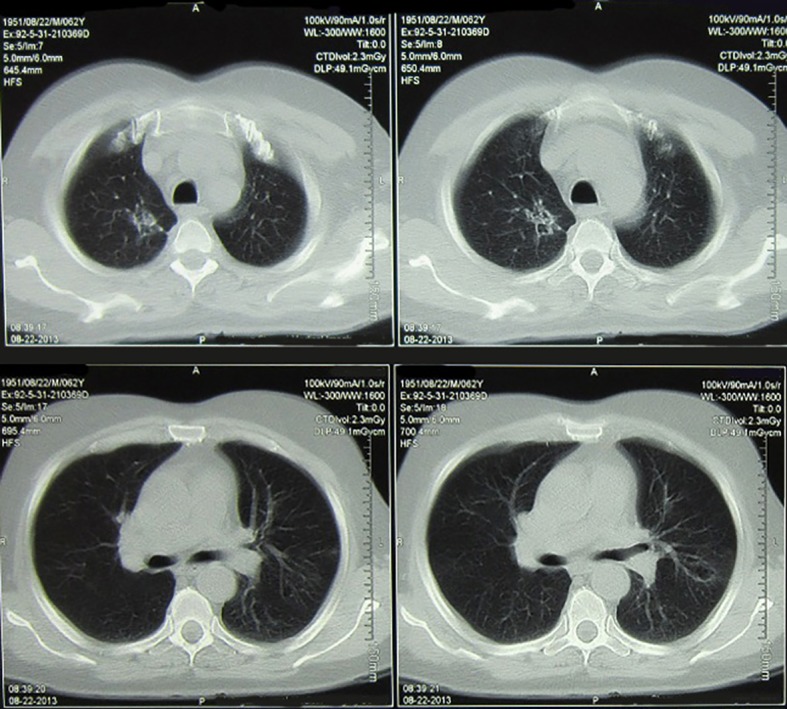
Shows the chest CT scan of the patient after the 3rd cycle of chemotherapy with cabazitaxel.

Therefore, chemotherapy with cabazitaxel 25 mg/m^2^ every three weeks with prednisolone 10 mg daily was started. After the third cycle of cabazitaxel, chest CT scan showed a complete recovery (figure 3).

Chemotherapy continued for three more cycles (with G-CSF support) and the treatment was completed in August 2013. The patient tolerated the chemotherapy with minor side effects, and eight months after the completion of the treatment, he was still symptom free. 
